# Anomaly Detection Using an Ensemble of Multi-Point LSTMs

**DOI:** 10.3390/e25111480

**Published:** 2023-10-26

**Authors:** Geonseok Lee, Youngju Yoon, Kichun Lee

**Affiliations:** Department of Industrial Engineering, College of Engineering, Hanyang University, 222 Wangsimni-ro, Seongdong-gu, Seoul 133-791, Republic of Korea; lgs5228@hanyang.ac.kr (G.L.); nanbread@naver.com (Y.Y.)

**Keywords:** anomaly detection, LSTM, ensemble technique

## Abstract

As technologies for storing time-series data such as smartwatches and smart factories become common, we are collectively accumulating a great deal of time-series data. With the accumulation of time-series data, the importance of time-series abnormality detection technology that detects abnormal patterns such as Cyber-Intrusion Detection, Fraud Detection, Social Networks Anomaly Detection, and Industrial Anomaly Detection is emerging. In the past, time-series anomaly detection algorithms have mainly focused on processing univariate data. However, with the development of technology, time-series data has become complicated, and corresponding deep learning-based time-series anomaly detection technology has been actively developed. Currently, most industries rely on deep learning algorithms to detect time-series anomalies. In this paper, we propose an anomaly detection algorithm with an ensemble of multi-point LSTMs that can be used in three cases of time-series domains. We propose our anomaly detection model that uses three steps. The first step is a model selection step, in which a model is learned within a user-specified range, and among them, models that are most suitable are automatically selected. In the next step, a collected output vector from M LSTMs is completed by stacking ensemble techniques of the previously selected models. In the final step, anomalies are finally detected using the output vector of the second step. We conducted experiments comparing the performance of the proposed model with other state-of-the-art time-series detection deep learning models using three real-world datasets. Our method shows excellent accuracy, efficient execution time, and a good F1 score for the three datasets, though training the LSTM ensemble naturally requires more time.

## 1. Introduction

Anomalies cause fatal problems in various systems, which can lead to the degradation of the system efficiency and performance. If anomalies are not detected on time, enormous losses can be incurred. In the case of a multi-variate time-series, it is difficult to ascertain anomalies with manual inspection, so a more accurate and faster anomaly detection algorithm is essential. The need for anomaly detection is increasing in various machine learning tasks, such as cyber-intrusion detection, the detection of intrusion into computer systems, fraud detection, the detection of illegal activities in insurance, credit, and financial data, malware detection, social network anomaly detection, industrial anomaly detection, and the detection of irregularities in industrial manufacturing data. Given the complex relationship among the data attributes, anomalies arise from not only one specific viewpoint but also various viewpoints. Since it is difficult to figure out anomalies in various fields with one model, many researchers have attempted to reflect the characteristics of various levels between the datasets.

In the past, anomaly detection relied on statistical and time-invariant methods. However, with the development of artificial neural networks and machine learning, various approaches for anomaly detection have been developed in recent years. For example, the application of such techniques permit the inclusion of temporal and contextual characteristics in the data that leads to a satisfactory anomaly-detection performance in complex systems.

Summarizing prior related research, we divide (classify) anomaly detection models into statistical, classification-based, clustering-based, and information-theoretic approaches. Thus, techniques based on the principal component analysis (PCA) [1], the support vector machine (SVM) [2], the k-nearest-neighbor algorithm [3], or different types of correlation analysis constitute a major part of earlier investigations. PCA is a representative type of unsupervised learning and is simultaneously the most representative dimension reduction algorithm. Since most real-life datasets share many features with each other, the use of these datasets is not efficient. PCA is an algorithm that reduces the data across dimensions with large correlation by projecting the data into a hyperplane. An important concept of the SVM is to find a plane that maximizes the margin between the data classes. This is called a decision boundary, and the data closest to the decision boundary is called a support vector. In the SVM algorithm, a class is classified by considering only the support vector. This is why SVM is a powerful algorithm for generalization. The KNN algorithm is a type of classification algorithm. In this approach, classification proceeds based on the distance between the datapoints according to the parameter k defined by the user. When k is too small, the noise is severe (the variance increases), and the classification is poor (bias increases). Therefore, it is important for the KNN algorithm to find an appropriate K value. The common ground for all of these approaches is their aim to detect anomalies based on static and time-invariant models [4]. For the detection of dynamic and time-varying anomalies, additional techniques, such as sliding windows, are utilized and combined with the aforementioned approaches. The approaches do not include models to adequately capture time-variant system dynamics, which leads to the unsatisfactory identification of anomalous phenomena. To tackle the problem of detecting complex contextual anomalies with dynamic and time-variant characteristics, researchers have adopted complex models. Among them, deep network architectures based on recurrent neural networks (RNNs) and their variants emerged. Many studies using RNN variants for anomaly detection have been conducted in recent years. For example, architectures using long short-term memory (LSTM) models have proved to be capable of solving a variety of complex detection tasks, as described in [5]. Indeed, many different deep neural network (DNN) or machine learning models using LSTM have been studied, such as LSTM-CNN [6], LSTM-SVM [7], LSTM-AE [8], and LSTM-VAE [9]. The LSTM-CNN extracts spatial characteristics for the adjacent data via a filter performing convolutional operations on convolutional layers sliding over sequences. Subsequently, the main feature is to make predictions by reflecting temporal characteristics via the LSTM network, an algorithm optimized for time-series information. In the case of LSTM-AE and LSTM-VAE, the autoencoder (AE) and variational autoencoder (VAE) do not consider temporal characteristics, so they are used with the LSTM to detect time-series anomaly detection. The LSTM-AE trains the characteristics of normal data. Since the LSTM-AE has learned only normal data, a large reconstruction error occurs when abnormal data is inputted. If the reconstruction error is larger than the predefined threshold, it is detected as abnormal data.

Early studies of ensemble learning conducted in the 1990s have demonstrated that several weak learning algorithms can be transformed into powerful algorithms [10,11,12]. By combining multiple weak classifiers with diverse viewpoints or strategies, ensemble methods can achieve a better generalization and accuracy. Recently, the ensemble LSTM models have proven to be efficient and successful in many applications such as stock prediction, intrusion detection, and network monitoring, achieving high classification accuracy and outperforming traditional LSTMs [13].

However, the effectiveness of aforementioned RNN or LSTM-based models has been validated usually for specific time-series scenarios. Therefore, our proposed method aims to work well in a general time-series domain. We take several different LSTM models that predict different responses to consolidate various views regarding an input time-series. By selecting top-performing LSTM models, we form an ensemble of the selected models, which we call multi-point LSTMs. We demonstrate that the proposed model of newly created multi-point LSTMs perform well in real-life anomaly detection tasks. The rest of this paper is organized as followed. We explain the background in Section 2 and introduce the proposed method in Section 3. We provide experimental results and show the effectiveness of the proposed method in Section 4. Section 5 concludes the paper with a description of future work.

## 2. Preliminaries

In this section, we briefly explain the long short-term memory model and its ensemble versions that are necessary for our newly proposed anomaly-detection model.

### 2.1. Long Short-Term Memory

However, RNN models suitable for time and sequences also have serious drawbacks. RNN models are severely degraded when they receive the input data with a long sequence [14]. As the length between the input data and the output data increases, the correlation usually decreases. This is called a long-term dependency problem. One of the models that aims to solve the long-term dependency of the RNN is LSTM [15]. The LSTM network is a very useful tool for predicting subsequent sequences by learning past and present self-dependent structures from the time sequence data. Also, a model called the gated recurrent unit (GRU) [16] is also a model that was developed to solve the long-term dependency problem of the RNN. The GRU is a simpler version of the LSTM structure. The LSTM model has three gates, but the GRU only has two gates. Therefore, the GRU can sometimes be faster than the LSTM model, but it shows a poor performance in situations with large datasets [17]. In this paper, we choose LSTM instead of the GRU because we want to show a good performance for both the large and small datasets.

**Figure 1:**
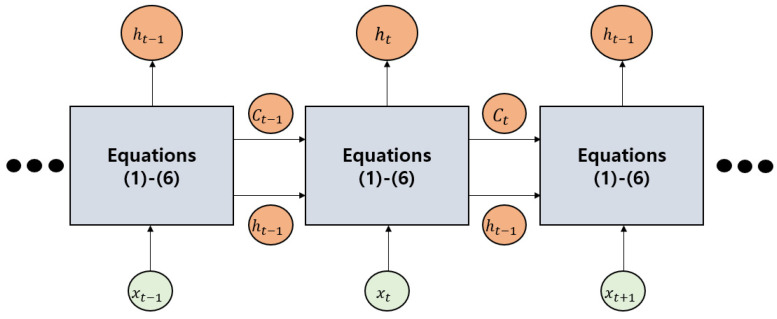
The structure of a long short-term memory model.

The layer structure of the LSTM model in contrast to the RNN is characterized by the presence of a state of cells shared between LSTM cells in addition to the output values. Figure 1 shows the structure of LSTM in which the cell ($C_{t}$) is shared. Different from RNN models, LSTM models share the cell state $C_{t}$, an additional state between the input layers. As these cell states are transferred to the next layer, they preserve the existing states to solve the problem of long-term dependency. There are three gates in the LSTM layer: input gate, forget gate, and output gate, to control the cell state value:

(1) $$i_{t}=\sigma \left(W_{i}x_{t}+U_{i}h_{t-1}+b_{i}\right)$$

(2) $${\tilde{C}}_{t}=\tanh\left(W_{C}x_{t}+U_{C}h_{t-1}+b_{C}\right)$$

(3) $$f_{t}=\sigma \left(W_{f}x_{t}+U_{f}h_{t-1}+b_{f}\right)$$

(4) $$C_{t}=f_{t}*C_{t-1}+i_{t}*{\tilde{C}}_{t}$$

(5) $$o_{t}=\sigma \left(W_{o}x_{t}+U_{o}h_{t-1}+b_{o}\right)≔LSTM\left(x_{t}\right)$$

(6) $$h_{t}=O_{t}*\tanh\left(C_{t}\right)$$

where $W_{i},W_{C},W_{o},W_{f},U_{i},U_{C},U_{o},U_{f},b_{i},b_{C},b_{o}$, and $b_{f}$ are the trainable parameters of the LSTM cell, σ is the sigmoid function, and ∗ is element-wise multiplication.

An input gate associated with Equations (1) and (2) is a gate that selects whether or not to store the current information. At this gate, we use the current input value $x_{t}$ and the previous hidden state $h_{t-1}$ to obtain the local state of the current cell and determine how much it reflects in the global cell state with the Sigmoid layer (1) and the tanh layer (2). Since the Sigmoid function is used, if the information obtained at this point is useful, it is highly reflected in the Cell State, otherwise, it is weighted close to zero to minimize the reflection.

The Equation (3) forget gate plays a role in deleting the data considered unnecessary among information from the past. Rather than precisely deleting the data, we multiply the weights between 0 and 1 obtained using the Sigmoid function (3) to have high weights for relatively important information while learning, and low weights for information that has a poor effect on the gradient updates. The algorithm multiplies the previous cell state $C_{t-1}$ by the forget gate output value $f_{t}$ and multiplies the output values from the input gate $i_{t}$ and the ${\tilde{C}}_{t}$ output of Equation (2) to update the state. Then, the previous two values are summed in a new cell state value. The Equation (5) output gate, which is the last gate, determines how much of the finally obtained cell state value $C_{t}$ is taken and transmitted to the hidden state. It is the sigmoid layer of the gate that determines which part of the cell state to output, which is calculated using Equation (5). The final value of $h_{t}$ given via Equation (6) moves back to the next cell state.

### 2.2. Ensemble LSTM

Ensemble models, traditionally built with different learners, have proven to be efficient for reducing the prediction variance and improving the accuracy. In an ensemble and boosting context, base learners can be any model working better than random ones, whether it be simple or complex [18,19,20]. Among many base-learner choices such as linear regression, kernel regression, spline, and network models, we choose a network model, LSTM, as a base learner. Indeed, to reflect diverse aspects in anomaly detection tasks, substantial researches on an ensemble LSTM have been conducted, such as using the dual LSTM [21], LSTM-based ensemble learning [22], DNN ensemble [23,24] AdaBoost-LSTM ensemble [25], and an ensemble of LSTM neural networks [26]. These papers are various extensions of LSTM models showing an excellent performance in time-series anomaly detection. However, the disadvantages of each model clearly exist. In the case of dual LSTM [21], two LSTMs are connected in series. Since the LSTM has a lower output dimension than the input dimension, it is difficult to use many LSTM ensembles in series. Therefore, it is unsuitable to produce an ensemble from many kinds of LSTM. The paper conducted in [22] proceeds with parallel learning for many LSTMs. This is useful for the data with characteristics by time zone, but inefficient in time-series data that continues regardless of the time features such as the heartbeat of a critically ill person or 24 h plant’s electricity volume. For a thesis [25], the AdaBoost-LSTM ensemble was constructed, which is also a model in which several LSTMs are trained in parallel. It is an algorithm that trains a number of N LSTMs to update the weights before producing an ensemble. At this time, when the hyper-parameter N increases, the computational complexity also increases excessively, and when N decreases to small values, the accuracy is reduced, since the weight update algorithm will not work well.

## 3. The Proposed Method: Ensemble of Multi-Point LSTMs

**Figure 2:**
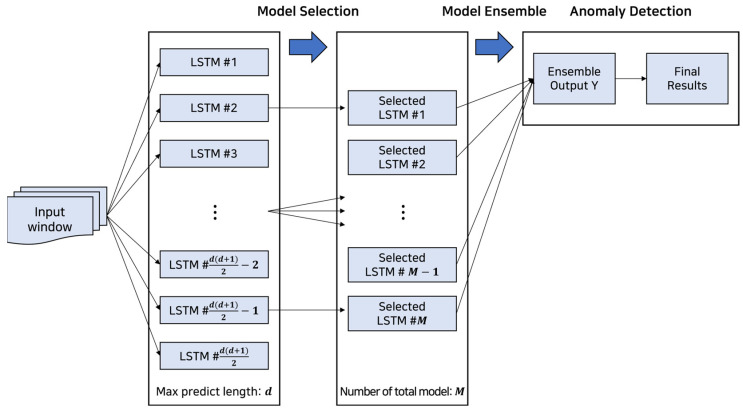
Steps of the proposed method, an ensemble of multi-point LSTMs.

In this section, we explain our proposed method, an ensemble of multi-point LSTMs, that generates numerous LSTM models reflecting diverse views and is equipped with a model selection and stacking procedure in training. Figure 2 shows the overall structure of our method. The proposed method, relying on the bagging concept, consists of three steps which are in-training model selection, model ensemble, and in-testing anomaly detection. In short, we represent multi-point LSTMs as LSTMs that predict different future time points with different lengths. Starting with the model parameters, a history point (h), and a max-prediction length (d), it forms an ensemble of multi-point LSTMs that are competitive and representative with different views.

Specifically, Algorithm 1 describes the whole process of the proposed method. First, let us define a vector of length h from time t in the input $x_{t}$ to be $x_{t:t+h}=\left[x_{t},x_{t+1},x_{t+2},\ldots ,x_{t+h-1}\right]$. The ${LSTM}_{n}^{k}\left(x_{t:t+h}\right)$ function, defined to be $o_{t}$ in Equation (5), is an LSTM model to predict the output ${\hat{Y}}_{t+h+n:t+h+n+k}$, that is to say, a signal starting an ‘n-step ahead’ time point with length k. The size of the LSTM output, its target for input $x_{t:t+h}$, is k, and n is the time-stamp gap between the end of $x_{t:t+h}$ and the start of the LSTM output. For example, ${LSTM}_{3}^{2}\left(x_{t:t+h}\right)$ aims to learn a three-step interval ahead of the signal of length two so that the output of $\hat{\left({LSTM}_{3}^{2}\right)}\left(x_{t:t+h}\right)$ may be close to ${\hat{Y}}_{t+h+3:t+h+5}=\left[{\hat{Y}}_{t+h+3},{\hat{Y}}_{t+h+4}\right]$.
**Algorithm 1.** Training and testing of an ensemble of multi-point LSTMs1:**In training**2:**Parameter:** d as max-prediction size3:**Input:** a collection of $x_{t:t+h}$ and ground-truth label $y_{t+h}\in \{0,1\}$, $t=1,\cdots ,T_{training}$4:**for** k=1 to d5:  **for** n=1 to d−k+16:    Learn ${LSTM}_{n}^{k}(x_{t:t+h})$, set it to $\hat{LSTM_{n}^{k}}\left(x_{t:t+h}\right)$, and compute ${MSE}_{k,n}$7:  
**end for**
8:**end for**9:**for** m=1 to $\frac{d(d+1)}{2}$10:  Find index set $\Gamma_{m}$ of (k,n) by selecting the smallest m ${MSE}_{k,n}$ among ${\left\{{MSE}_{k,n}\right\}}_{allk,n}$11:  Create a concatenated input vector as $X_{t}={\left[\hat{LSTM_{n}^{k}}\left(x_{t:t+h}\right)\right]}_{\left(k,n\right)\in \Gamma_{m}}$12:  Learn decision tree ${\hat{f}}_{m}$ using $X_{t}$ and $y_{t+h}$ for all t and compute accuracy $acc_{m}$13:**end for**14:Set M by finding the largest accuracy: $M=argmax{\left\{acc_{m}\right\}}_{m=1,\cdots ,\frac{d(d+1)}{2}}$15:**Output:** ${\hat{f}}_{M}$16:**In testing**17:**Input:** a new time series, $x_{t_{new}:t_{new}+h}$, starting at time stamp $t_{new}$ of length h18:${\tilde{Y}}_{t_{new}+h}={\hat{f}}_{M}({\left[\hat{LSTM_{n}^{k}}\left(x_{t_{new}:t_{new}+h}\right)\right]}_{\left(k,n\right)\in \Gamma_{M}})$19:**Output:** normal(0) or anomaly(1) using ${\tilde{Y}}_{t_{new}+h}$

The input of Algorithm 1 is a ground-truth collection of the length-h signal, $x_{t:t+h}$, and its corresponding label $y_{t+h}\in \left\{0,1\right\}$ from $t=1,\ldots ,T_{training}$ with $y_{t+h}$ being a binary label, meaning normal or anomaly, for the input signal $x_{t:t+h}$. The output of Algorithm 1 in training is a final anomaly detector as an ensemble of several ${LSTM}_{n}^{k}\left(x_{t:t+h}\right)$ models, and that of Algorithm 1 in testing is a prediction of anomaly existence associated with the input $x_{t_{new}:t_{new}+h}$. Lines 4 through 13 and lines 14 through 15 correspond to the model selection and the model ensemble, respectively. The parameter, d, in line 2 represents the max-prediction length for which the prediction of the candidate LSTM models starts from t+h+1 up to t+h+d, as shown in lines 4 to 8. By constructing d(d+1)/2 number of candidate LSTM models, via iterations by k and n, as implemented in lines 4 to 5, we choose the top M models among them in terms of the mean square error (MSE). Thus, the time complexity is $O\left(d^{2}\right)$, but noticeably, the two iterations by k and n can proceed in a parallel manner since no dependence exists in the cell execution on lines 9 to 15 as for k and n. After the 16th line, it is a process of testing with a new input $x_{t_{new}:t_{new}+h}$ using ${\hat{f}}_{M}\left({\left[\hat{\left({LSTM}_{n}^{k}\right)}\left(x_{t_{new}:t_{new}+h}\right)\right]}_{\left(k,n\right)\in \Gamma_{M}}\right)$, which is made up by the learned multi-point LSTMs. We provide a detailed description in the following sections.

### 3.1. Model Selection

In the first stage, with an aim to include numerous LSTM models with different aspects, we train all possible LSTMs with prediction length ranges from one to d (the max-predict size). It is implemented via iteration by n on line 5 in Algorithm 1, where n ranges from 1 to d−k+1. For example, when the prediction-gap k is 1, the algorithm learns a number d of LSTM models to predict ${\hat{y}}_{t+h+1:t+h+2},{\hat{y}}_{t+h+1:t+h+3},\cdots ,{\hat{y}}_{t+h+1:t+h+1+d}$, respectively, for the given $x_{t:t+h}$. In addition, we make the k-step ahead forecast in the construction of possible LSTMs, where k ranges from 1 to d. It is implemented via iteration by k on line 4 in Algorithm 1. For example, n = 1 means that the algorithm prepares a number d of LSTM models to predict ${\hat{y}}_{t+h+1},{\hat{y}}_{t+h+2},\mathrm{and}{\hat{y}}_{t+h+1+d}$, respectively, for the given $x_{t:t+h}$.

Suppose we set the max predict size d to 10 for the given input data $x_{t:t+h}$. In the training session, we train 10 LSTMs for k=1, which produce 10 output vectors $\hat{\left({LSTM}_{1}^{1}\right)}\left(x_{t:t+h}\right),\hat{\left({LSTM}_{2}^{1}\right)}\left(x_{t:t+h}\right),\ldots ,\hat{\left({LSTM}_{10}^{1}\right)}\left(x_{t:t+h}\right)$ to predict $y_{t+h+1},y_{t+h+2},\ldots ,y_{t+h+10}$, respectively. Then, for k=2, we train nine LSTMs predicting two intervals sequentially: the LSTM outputs are nine vectors in dimension 2-by-1, such as $\hat{\left({LSTM}_{1}^{2}\right)}\left(x_{t:t+h}\right)=\left[{\hat{y}}_{t+h+1},{\hat{y}}_{t+h+2}\right],\hat{\left({LSTM}_{2}^{2}\right)}\left(x_{t:t+h}\right)=\left[{\hat{y}}_{t+h+2},{\hat{y}}_{t+h+3}\right],\ldots ,\hat{\left({LSTM}_{9}^{2}\right)}\left(x_{t:t+h}\right)=\left[{\hat{y}}_{t+h+9},{\hat{y}}_{t+h+10}\right]$. Likewise, for k=3, we obtain eight output vectors in dimension 3-by-1 such as $\hat{\left({LSTM}_{1}^{3}\right)}\left(x_{t:t+h}\right)={\hat{y}}_{t+h+1:t+h+4},\hat{\left({LSTM}_{2}^{3}\right)}\left(x_{t:t+h}\right)={\hat{y}}_{t+h+2:t+h+5},\ldots ,\hat{\left({LSTM}_{7}^{3}\right)}\left(x_{t:t+h}\right)={\hat{y}}_{t+h+7:t+h+10}$. By repeatedly predicting up to the max predictor size d=10, we finally obtain one 10-by-1 dimensional output vector for k=10, $\hat{\left({LSTM}_{1}^{10}\right)}\left(x_{t:t+h}\right)={\hat{y}}_{t+h:t+h+10}$, whose output vector length is the max predictor size. In this way, we train 55 different LSTMs for d=10; in general, we train (d(d+1))/2 different LSTMs. Our view is that the inclusion of all sequential and subsequent time-stamps will be sufficient to reflect possible pattern changes associated with the input $x_{t:t+h}$. If we do not make predictions sequentially with k=3, for example, considering an LSTM model to predict $\left[{\hat{y}}_{t+h+1},{\hat{y}}_{t+h+4},{\hat{y}}_{t+h+9}\right]$, we need to train as many as $2^{10}$ models. The computational complexity increases to $2^{d}$ in general. On the other hand, our method has a low computational complexity of $O\left(d^{2}\right)$, reflecting all sufficient points considering the time-series sequences.

As a tuning parameter d, we recommend the setting of d to be neither too small nor too large, so that it may include the resulting fluctuations and pattern-changes right after receiving the input signal $x_{t:t+h}$ of size h. We proceed with the experiment to recommend the proper values of the parameters h and d.

### 3.2. Model Ensemble: Stacking Ensemble

Ensemble learning is a procedure for extracting multiple predictions by applying multiple learner modules to the datasets and combining them into one composite prediction. In general, two steps are used. In the first step, a set of base learners are obtained from the training data, and in the second step, the learners obtained in the first step are combined in order to produce a unified prediction model. Following the ensemble principle, in this research, we train a stacking ensemble to build one unified model using various LSTM models for ultimate predictions. We notice that the adopted stacking ensemble is the most suitable ensemble algorithm in the case of the regression problem [27] and it has been successfully applied for solving pattern classification, regression, and forecasting in time-series problems [28,29].

### 3.3. Anomaly Detection

In the last stage, we finally detect anomalies with a classification model, a decision tree [30]. The collected output vector from M LSTMs, denoted via index set $\Gamma_{M}$ in Algorithm 1, obtained in the previous stage is used as the input data $X_{t}$ of a decision tree to determine whether the original input data $x_{t:t+h}$ is normal or anomalous. We concatenate the M output vectors via $X_{t}={\left[\hat{\left({LSTM}_{n}^{k}\right)}\left(x_{t:t+h}\right)\right]}_{\left(k,n\right)\in \Gamma_{M}}$ from the automatically determined M LSTMs. We view the concatenated vector, $X_{t}$, as a new embedding and representation for the original input data $x_{t:t+h}$. In Algorithm 1, additionally, a suitable decision tree ${\hat{f}}_{M}$ is determined in line 14, and lines 16 to 19 are the process of classifying the final output, normal and anomalous, using the decision tree, ${\hat{f}}_{M}$. Tree-based models are often used for binary classification. Among several machine learning models available, such as random forests and support vector machines, we choose to use a decision tree model since the LSTM outputs are already selectively collected and unified. We notice that the classifier choice makes little difference in outlier detection tasks, but it demonstrates the practical performance of the decision tree in the experiment section.

## 4. Experiments

In this section, we provide experimental results to compare the performance of the proposed method with the selected anomaly-detection models.

### 4.1. Datasets

We used three real-life datasets with different numbers of observation points, numbers of features, and anomaly rates. MobiAct is a publicly available dataset that includes the data from a smartphone when participants are performing different types of activities and experience a range of falls. It is based on the previously released MobiAct dataset [31]. The MobiAct dataset included a total of 66 subjects’ daily life scenarios through four different types of falls (anomalies) and 12 different activities of daily living (normal) with more than 3200 trials, all measured with smartphones and smartwatches. The dataset was collected with six features of the XYZ-axis accelerometer and XYZ-axis gyroscope, and a total of 565,599 observations were used. Among them, the number of abnormal observation points is 145,444, and the abnormal rate is 34.61%.

The Water Distribution (WADI) [32] testbed, funded by the SUTD-MIT International Design Centre (IDC), was launched on 26 July 2016 by Cyber Security Agency’s (CSA) Deputy Chief Executive Mr Teo Chin Hock. The dataset was collected for 16 days and cyber attacks were carried out for 2 days. The number of features is 93, and we used 17,280 observations. The abnormal rate is 6.10%, which is the lowest among the three datasets.

The Secure Water Treatment (SWaT) [33] testbed is a test bed produced in 2015 by SUTD’s iTrust Research Institute, which was built by analyzing a typical water treatment system used in a city and consists of a total of six stages. The dataset is captured without stopping for a total of 11 days, where 7 days are the normal operation data, and the remaining 4 days are the data including the cyber attack execution. The dataset was collected with a total of 51 features and we used 395,919 observations. The number of abnormal datapoints is 51,483, and the abnormal rate is 14.94%. The summary is shown in Table 1.

**Figure 3:**
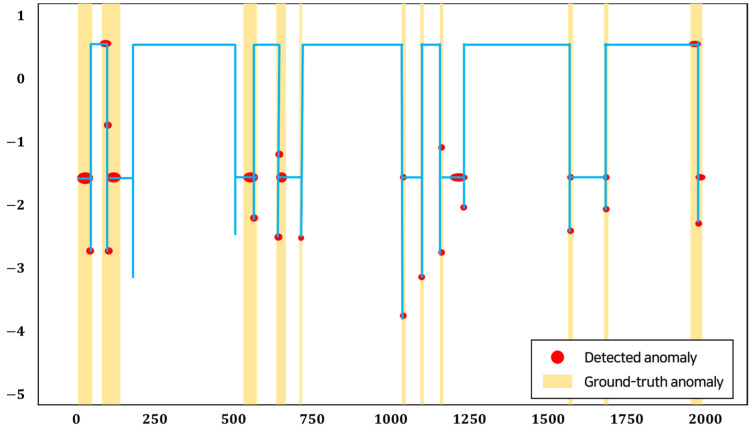
Illustration of anomaly detection in the SWaT dataset.

Figure 3 is an illustration of the performance of our model on the SWaT dataset that detects anomalies. The SWaT dataset used in Figure 3 was normalized for visualization. The SWaT dataset, containing 51 features, includes 14.94% anomalies. It shows our method not only detects outliers that differ greatly from the normal values, but also detects anomalies similar to the normal values from a univariate perspective, though it used 51 multivariate features. The values indicated by ‘x’ in red are the detected anomaly, and the values indicated by the blue circle are the ground-truth anomalies.

### 4.2. Evaluation Metrics

Since anomaly detection is a binary classification task, we evaluate the performance with two evaluation criteria, namely accuracy and the F1 score, as in other research [34,35]. Using the true positive (TP), true negative (TN), false positive (FP), and false negative (FN) rates, we define the accuracy as

$$Accuracy=\frac{TP+TN}{TP+TN+FP+FN}$$

Accuracy refers to the probability of correct answers among all classified observations. We also use the F1 score as a performance evaluation indicator in this research. We compute the F1 score as follows:

$$F1-score=\frac{2\times (Precision\times Recall)}{Precision+Recall}$$

where Precision=TP/(TP+FP) and Recall=TP/(TP+FN). The precision n is the ratio of what the model correctly classifies as true to the sum of true classifications and erroneous true classifications, and the recall is the ratio of what the model predicts to be true over the sum of accurate true classifications and inaccurate negative classifications. The F1 score is the harmonic mean of precision and recall, which is an effective performance indicator when a dataset is unbalanced.

### 4.3. Experimental Results

We change various conditions to set the hyper-parameters of the input window (h), max predict size (d), and the number of final models (M) in Table 2, which shows the performance in terms of the F1-score. The test data used in the experiment does not overlap with the training data used in learning. Notice that we used datasets for testing that are not within its training session for the performance computation. The time interval of the MobiAct dataset is 0.2 s, and the time intervals of the SWaT and WADI dataset are 10 s. The input window size should be determined by considering the time interval of the input window. WADI and SWaT, which are datasets with large time intervals, tend to slightly underperform when the input window size is large, and MobiAct, which is a dataset with small time intervals, performs better when receiving a larger input window size as the input. Also, considering the number of features and the lengths of each dataset, the input window for MobiAct is set to 20, and those for WADI and SWaT are set to 10. The max predict size was set to 20 for the three datasets, and the number of final models (M) that showed the best performance were automatically determined to be 12, 9, and 13, for the respective MobiAct, WADI, and SWaT datasets. In Table 2, we observe that the performance of the model increases as d increases. However, one needs to be cautious in that a large d causes the model to be slow in both training and testing, which we will show in the following section. In consideration of the computing situation, we conducted the experiment for d up to 20.

To report the performance with simplicity, we denote our proposed model ‘Multi-point LSTMs’ as M-LSTMs. We compare four machine learning models, namely random forest (RF), xgboost, support vector machine (SVM) [2], and logistic regression, in the construction of anomaly detection models. In addition, we use four deep learning models, namely LSTM [21], LSTM-AE [8], LSTM-VAE [9], and LSTM-CNN [6], as comparative models.

In Table 3, the ‘M-LSTMs’ show the highest performance in accuracy and the F1 score. It shows a good performance in the order of LSTM-CNN and LSTM, following M-LSTMs, in terms of accuracy. Overall, the F1 scores are high because the anomaly rate is the highest among the three datasets. Since the ratio of normal to abnormal data is about 2:1, the machine learning models also produced F1 scores that were generally low for most models.

The WADI dataset has the highest number of features and the lowest anomaly rate among the three datasets. Although the M-LSMTs model yielded the second-lowest lower F1 following the LSTM-CNN model, our model shows a higher accuracy than LSTM-CNN as shown in Table 4. We suspect that training the model with sufficient epochs in a better computing environment would increase its performance for this dataset. Since the anomaly rate is quite low, machine learning models show quite poor F1 score results.

In the experiment with the WADI dataset, our proposed model, shows the best results in terms of both accuracy and the F1 score. In particular, the result of the F1 score shows a value that is 0.0875 higher than the second highest value of LSTM-CNN. Similarly, in the experiment with the SWaT dataset, the proposed model yields the best performance in both measures, as shown in Table 5. From these experiments, we observe that the datasets with a large number of observation points and with a high anomaly rate produce a satisfactory performance.

### 4.4. Further Results

In this section, we compare the performance of the classification models using a decision tree, random forest, and support vector machine in the final ‘anomaly detection’ stage. We also compare the experimental training and execution times of the proposed model and the adopted models operating on the three datasets.

Table 6 shows the performance results depending on the classification models. A random forest is a method of using several ensemble decision trees. In addition, since it compensates for the shortcomings of decision trees, the accuracies of both the decision tree and random forest models are generally high and greatly correlated. However, using the SWaT dataset, the decision tree outperformed the random forest in accuracy. In addition, we opted to use the decision tree because the random forest method requires a longer learning time than the decision tree, and it is difficult to interpret as a purely black box model. The SVM shows the lowest performance in this experiment, which serves as a bottom-line performance because the performance of the SVM is greatly influenced by the hyper-parameter margin value and the kernel choice.

**Figure 4:**
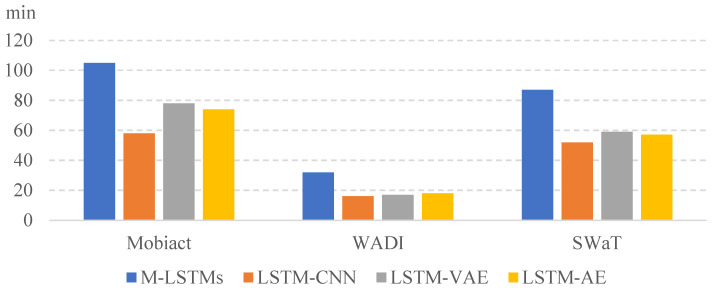
Training time of the selected deep learning models.

**Figure 5:**
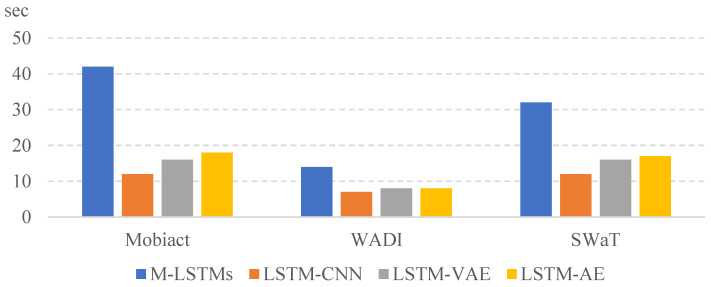
Testing time of the selected deep learning models.

Figure 4 and Figure 5 show the results of the training and testing time measured for the three datasets, Mobiact, SWaT, and WADI. The training time for each model was long to short in the order of M-LSTMs, LSTM-VAE, LSTM-AE, and LSTM-CNN. Depending on the computation complexity, if d is doubled, the learning time is about four times longer. The unit of training time is a minute and that of the testing time is a second. We split 70% of the total data for training and used the remaining 30% for testing. The computing settings of our all experiments are two RTX 3090 GPU cards with 128 GB RAM using Tensor Flow version 2.8.0. We notice that the M-LSTM runs took the longest to train, which is naturally understood because the model builds an ensemble of neural networks in its training. However, we also notice that the absolute in-total testing run times of the models are not significantly different, with all executions taking place in less than a minute. This means that the proposed model, once built, runs quite fast compared with the other models. This is important since most computational overhead in a real-world application will be devoted to testing the real-time data for anomalies.

## 5. Conclusions

In this paper, we propose a time-series anomaly detection model that shows the performance improvement for three different cases of datasets. The proposed model is a model that produces an ensemble of multiple LSTM results to make decisions, which can be used more broadly because the appropriate LSTM for the domain is selected based on real-world data. The proposed multi-point LSTM model selection has three steps to detect anomalies. First, in the model selection step, we train a myriad sequence of parallel LSTM models. Among them, m models with a good performance are selected and sent to the next step. The outputs of the m successful LSTM models are automatically combined into a stacked ensemble and are reconfigured for the final neural network outputs. In the last step, we use a decision tree to detect outliers. In the last step, the final neural network output calculated in the previous step is used as the input to the decision tree to detect anomalies.

We conduct the experiment with three different real-world datasets, MobiAct, WADI and SWaT. The three datasets had very different observation points, number of features, and anomaly rates. Working without hand-tailoring the model to the real-world dataset, our proposed model showed a good performance in a robust manner. Although our model requires a long learning time compared with other state-of-the-art models, it can be said to be a more suitable model for anomaly detection situations that require high accuracy regardless of the learning time. It should be noted that the proposed method applied to the WADI dataset had a slightly lower F1-score than the comparative model LSTM-CNN, since this method will show a better performance if the learning is deepened, given the superior computing power or given the longer learning times. Our model achieved its goal of adaptive anomaly detection regardless of the input data across all tested datasets.

The proposed method is designed to detect anomalous patterns in time-series using LSTM ensemble models. However, ensemble methods suffer from an inherent limitation of constructing several deep learning models, which incur high computational costs. Our method chose a ‘black-box’ network model as the base learner to utilize its performance-sacrificing interpretability. Therefore, further research will focus on processing the real-time data and allowing the online update of the learned model. In addition, in the future, we will study a model that automatically determines the optimal parameter values for d, h, and M. We will observe the effect of different base learners, starting from a simple one for the sake of interpretability, other than the network model adopted in the method. We will also envision investigating the generalization performance in the samples unseen or different from the in-training ones.

## Figures and Tables

**Table 1:** Dataset summary.

Dataset	MobiAct	WADI	SWaT
Number of features	6	93	51
Number of observations	565,599	17,280	395,919
Number of normalcy	420,155	16,285	344,436
Number of anomaly	145,444	995	51,483
Anomaly rate	34.61%	6.10%	14.94%

**Table 2:** Performance according to hyper-parameters.

Dataset	MobiAct			WADI			SWaT		
	d = 10	d = 15	d = 20	d = 10	d = 15	d = 20	d = 10	d = 15	d = 20
h = 10	0.8970	0.8989	0.9063	0.6121	0.6173	0.6213	0.9273	0.9299	0.9387
h = 20	0.9135	0.9196	0.9222	0.5892	0.5984	0.6057	0.9092	0.9124	0.9166
h = 30	0.9092	0.9137	0.9178	0.5998	0.5991	0.6088	0.8698	0.8801	0.8891

**Table 3:** Model performances using the MobiAct dataset.

Dataset	Model	Accuracy (%)	F1 Score
Mobiact	RF	75.96	0.6965
	Xgboost	74.32	0.6679
	SVM	68.61	0.6255
	Logistic Regression	65.93	0.5973
	LSTM	92.10	0.8589
	LSTM-AE	81.35	0.6057
	LSTM-VAE	84.32	0.7568
	LSTM-CNN	93.10	0.8689
	M-LSTMs	**95.87**	**0.9222**

**Table 4:** Performance on the WADI dataset.

Dataset	Model	Accuracy (%)	F1 Score
WADI	RF	73.04	0.2893
	Xgboost	75.79	0.2991
	SVM	79.44	0.3240
	Logistic Regression	78.19	0.3222
	LSTM	88.39	0.5831
	LSTM-AE	82.10	0.5367
	LSTM-VAE	86.88	0.5534
	LSTM-CNN	92.10	**0.6241**
	M-LSTMs	**92.17**	0.6213

**Table 5:** Model performances on the SWaT dataset.

Dataset	Model	Accuracy (%)	F1 Score
SWaT	RF	70.96	0.4956
	Xgboost	72.20	0.5284
	SVM	64.31	0.4905
	Logistic Regression	67.29	0.5007
	LSTM	92.95	0.8289
	LSTM-AE	92.26	0.7857
	LSTM-VAE	94.88	0.8068
	LSTM-CNN	96.29	0.8512
	M-LSTMs	**97.66**	**0.9387**

**Table 6:** Performance depending on different classification models.

Dataset	Model	Accuracy (%)	F1 Score
MobiAct	Decision Tree	95.87	**0.9222**
	Random Forest	**96.12**	0.9135
	SVM	94.25	0.9016
WADI	Decision Tree	92.17	**0.6213**
	Random Forest	**94.28**	0.6129
	SVM	85.67	0.5296
SWaT	Decision Tree	**97.66**	**0.9387**
	Random Forest	96.13	0.9224
	SVM	94.08	0.9120

## Data Availability

Not applicable.
